# Collagen Dressing Versus Conventional Dressings in Burn and Chronic Wounds: A Retrospective Study

**DOI:** 10.4103/0974-2077.79180

**Published:** 2011

**Authors:** Onkar Singh, Shilpi Singh Gupta, Mohan Soni, Sonia Moses, Sumit Shukla, Raj Kumar Mathur

**Affiliations:** *Department of Surgery, MGM Medical College and MY Hospital, Indore, India*

**Keywords:** Burns, chronic wounds, collagen dressing

## Abstract

**Objective:**

Biological dressings like collagen are impermeable to bacteria, and create the most physiological interface between the wound surface and the environment. Collagen dressings have other advantages over conventional dressings in terms of ease of application and being natural, non-immunogenic, non-pyrogenic, hypo-allergenic, and pain-free. This study aims to compare the efficacy of collagen dressing in treating burn and chronic wounds with that of conventional dressing materials.

**Materials and Methods:**

The records of 120 patients with chronic wounds of varied aetiologies and with mean age 43.7 years were collected and analyzed. The patients had been treated either with collagen or other conventional dressing materials including silver sulfadiazine, nadifloxacin, povidone iodine, or honey (traditional dressing material). Patients with co-morbidities that could grossly affect the wound healing like uncontrolled diabetes mellitus, chronic liver or renal disease, or major nutritional deprivation were not included. For the purpose of comparison the patients were divided into two groups; ‘Collagen group’ and ‘Conventional group’, each having 60 patients. For assessment the wound characteristics (size, edge, floor, slough, granulation tissue, and wound swab or pus culture sensitivity results) were recorded. With start of treatment, appearance of granulation tissue, completeness of healing, need for skin grafting, and patients’ satisfaction was noted for each patient in both groups.

**Results:**

With two weeks of treatment, 60% of the ‘collagen group’ wounds and only 42% of the ‘conventional group’ wounds were sterile (*P*=0.03). Healthy granulation tissue appeared earlier over collagen-dressed wounds than over conventionally treated wounds (*P*=0.03). After eight weeks, 52 (87%) of ‘collagen group’ wounds and 48 (80%) of ‘conventional group’ wounds were >75% healed (*P*=0.21). Eight patients in the ‘collagen group’ and 12 in the ‘conventional group’ needed partial split-skin grafting (*P*=0.04). Collagen-treated patients enjoyed early and more subjective mobility.

**Conclusion:**

No significant better results in terms of completeness of healing of burn and chronic wounds between collagen dressing and conventional dressing were found. Collagen dressing, however, may avoid the need of skin grafting, and provides additional advantage of patients’ compliance and comfort.

## INTRODUCTION

During the last decade, various new dressing materials developed, like calcium alginate, hydro-colloid membranes and fine mesh gauze. These have a disadvantage in that they become permeable to bacteria. Biological dressings like collagen on the other hand, create the most physiological interface between the wound surface and environment, and are impermeable to bacteria.[1] Collagen dressings have other advantages over conventional dressings in terms of ease of application and being natural, non-immunogenic, non-pyrogenic, hypo-allergenic, and pain-free.[2,3] The present study has been conducted to compare the efficacy of collagen dressing with that of conventional dressing materials like silver sulfadiazine, nadifloxacin, povidone iodine, or honey (used traditionally), in the management of chronic wounds including those due to burns.

## MATERIALS AND METHODS

We retrospectively collected the records of the patients with chronic wounds on different parts of the body and of various aetiologies, treated in our department with either collagen dressings or one of the conventional dressing materials/honey, over a period of four years (2006-2009). The total number of patients was 120. The patients with co-morbidities that could grossly affect the wound healing like uncontrolled diabetes mellitus, chronic liver or renal disease, other collagen disease or major nutritional deprivation were excluded. For the sake of analysis the patients were divided into two groups; ‘Collagen group’ and ‘Conventional group’. The recorded data from the patients’ files regarding characteristics of all wounds as size, edge, floor characteristics, slough, granulation tissue, pathogenic organisms and wound swab or pus culture sensitivity results were noted and analyzed. Wound swab or pus culture was done every three to five days or when specifically required (hospital protocol).

Before applying collagen dressing, the affected area was thoroughly cleaned for removal of external contamination, and infected wound was debrided properly. Then, one or more collagen sheets (manufactured from intestine of cattle by The Central Leather Research Institute, Adyar, Chennai) of appropriate size are selected. Collagen sheets were rinsed in normal saline before application. Sheets were applied firmly so as to cover the whole raw area of wound/ulcer. Care should be taken to remove any air bubbles. This can be facilitated by using the back of the thumb-forceps to apply a little pressure from one end of the dressing to the other. The movement of the forceps should be just similar to the movement of a knife while applying butter on a toast. Dressing was then dried with a warm-air dryer.

Wounds of the patients in the ‘Conventional group’ were dressed with povidone iodine, honey, nadifloxacin, or silver sulfadiazine etc. Both the groups were treated with antibiotics based on the pus culture sensitivity report. Response to the treatment and patients’ outcome were noted in terms of progression of wound healing, granulation tissue formation, changes in edges of wounds and need of skin grafts. The results were analyzed using ‘Mann-Whitney test’ and ‘Pearson Chi-Square test’ depending on the type of data.

## RESULTS

A total of 120 patients were included. Seven different aetiologies of chronic wounds were recognized: decubitus ulcer, post-traumatic wound, venous ulcer, post-burn, postoperative, post-infection, and miscellaneous. Out of 120 patients, 24 (20%) belonged to the age group 01-20 years, 68 (57%) to 20-40 years, and 28 (23%) were more than 40 years of age. Eighty-two (68%) were males and 38 (31%) were females. There was no significant difference in the age and sex distribution of patients and aetiology of the wounds in both groups [Table 1].

**Table 1:** Characteristics of the patients in the ‘Collagen Group’ and ‘Conventional group’

Patient characteristics		Collagen Group (n=60)		Conventional Group (n=60)		*P* value
		No. of Cases	%	No. of Cases	%	
Sex	Male	42	70	40	67	0.9*
	Female	18	30	20	33	
Type of wound	Decubitus	12	20	9	15	0.67**
	Post-traumatic	5	8	4	7	
	Venous	3	5	3	5	
	Post-burn	16	27	20	33	
	Postoperative	7	11	3	5	
	Post- infection	13	22	16	27	
	Miscellaneous	4	7	5	8	
Age	01-20 yrs	15	25	9	15	0.35*
	20-40 yrs	31	52	37	62	
	>40 yrs	14	23	14	23	

* -Mann-Whitney test

** -Pearson Chi-Square test

The most common pathogens found on wound swab cultures (taken from three different sites in all patients) of patients with burn, postoperative, venous ulcers and post-traumatic wound/ulcers were *Staphylococcus* and *E. coli*. Decubitus and post-infectious ulcers were mostly infected by *E. coli* and *Pseudomonas*. Overall *Staphylococcus* was the pathogen most often isolated (45%), followed by *E. coli* (20%), *Pseudomonas* (20%) and *Klebsiella* (8.3%). Sixty percent of the ‘collagen group’ wounds showed complete clearance of organisms within two weeks, 90% (54) in four weeks while only six wounds did not show clearance of organisms at the end of four weeks. On the other hand, only 42% of the wounds in the ‘conventional group’ were found sterile after two weeks of treatment. After four weeks of conventional treatment 12 (20%) wounds were still found to harbour pathogenic organisms [Table 2].

**Table 2:** Response to the treatment

Treatment response	Collagen Group (n=60) %		Conventional Group (n=60) %		*P*-value
Sterile wound swab culture at two weeks	36	60	25	42	0.03*
Sterile wound swab culture at four weeks	54	90	48	80	0.04
Avg. time for healthy granulation to appear	8 days		14 days		0.03**
Complete wound closure at six weeks	42 (70)		38 (63)		0.22*
At eight weeks, wounds with >75% closure	52 (87)		48 (80)		0.21*
Wounds that required SSG	8 (13)		12 (25)		0.04*

* -Mann-Whitney test

** -Pearson Chi-Square test; Figures in parenthesis are in percentage

The average time for appearance of healthy granulation tissue over the wounds that were treated with collagen dressing was eight days. The post-infective wounds healed fastest (average time to healthy granulation tissue: six days) and the decubitus wounds slowest (average time to healthy granulation tissue: 13 days).

In the ‘conventional group’ the average time for appearance of healthy granulation tissue was 14 days. In this group, the post-traumatic wounds healed fastest (average time to healthy granulation tissue: eight days) and decubitus wounds slowest (average time to healthy granulation tissue: 16 days) [Table 2]. When the wounds of the patients in both groups were compared aetiology-wise, each type of wound treated with collagen dressing had lesser average time for appearance of healthy granulation tissue, than the same type of wound treated with conventional dressing material.

**Figure 1:**
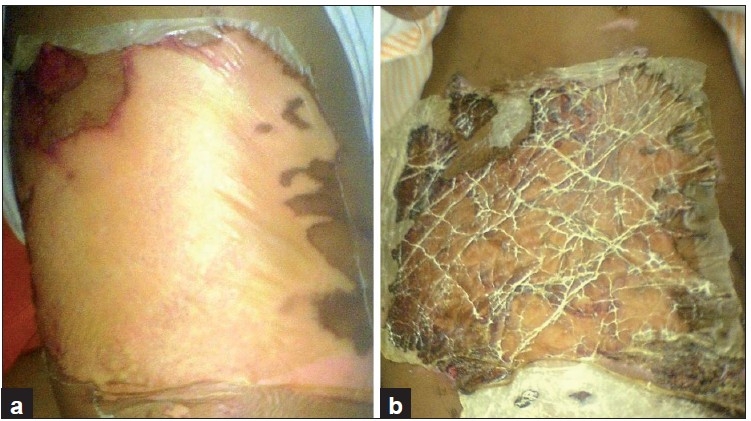
A superficial burn wound on day one (a) and on 21^st^ day (b) of collagen dressing

**Figure 2:**
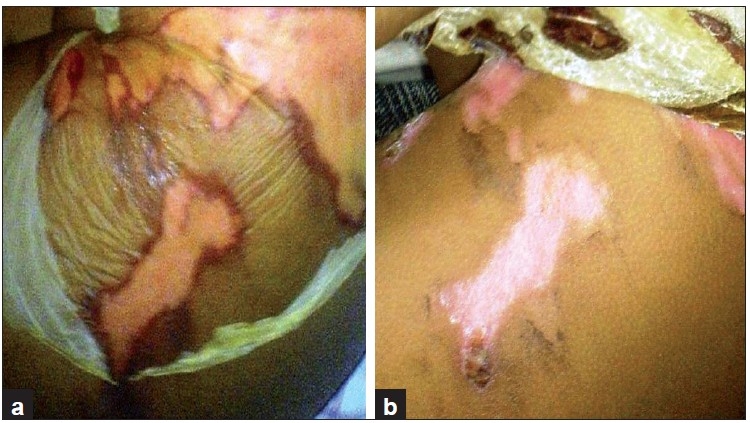
A superficial wound with applied collagen dressing on day one (a) and after three weeks (b) Collagen dressing being easily removed

**Figure 3:**
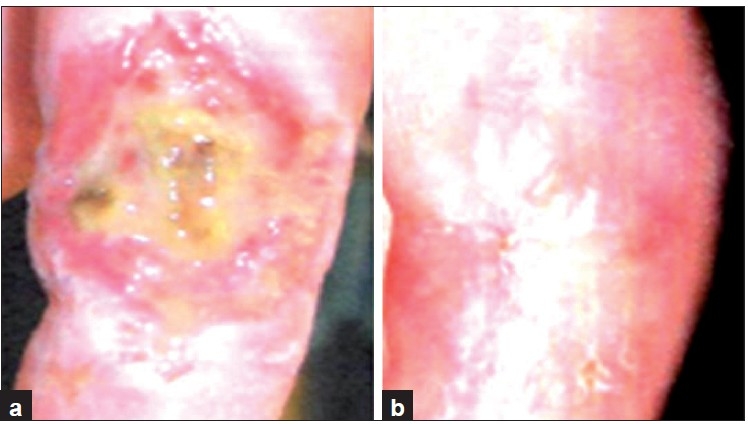
A deep wound on leg before (a) and after 14 days (b) of collagen dressing

**Figure 4:**
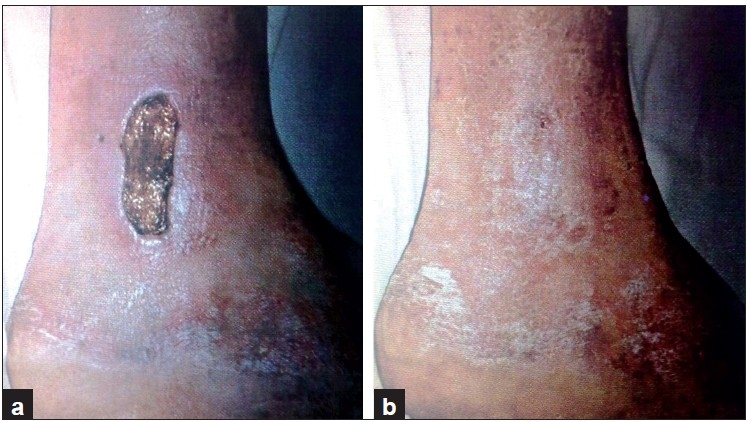
A deep wound on leg before (a) and after 28 days (b) of collagen dressing

**Figure 5:**
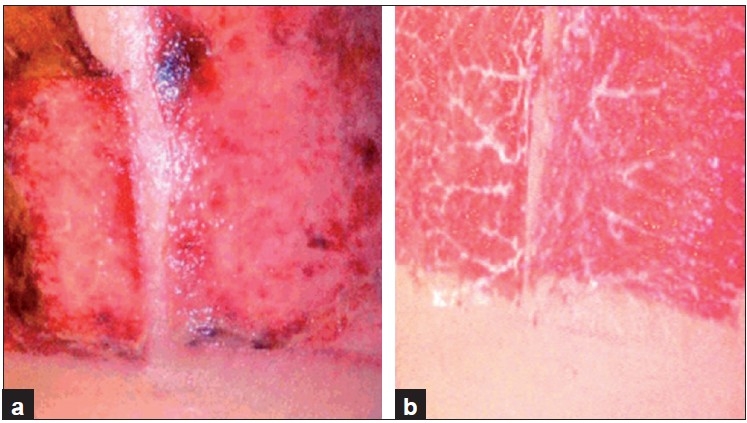
Another deep wound on the back before (a) and after a week (b) of collagen dressing

It was found that out of 60 patients of the collagen group, 42 (70%) wounds showed complete closure with collagen dressing [Figures 1–4] in six weeks or lesser time, and 10 more wounds showed 75-100% closure in the next two weeks [Figure 5 a and b]. Collagen sheets in these patients were found almost fully incorporated in the wounds. The remaining eight (four-decubitus, four-post-burn) achieved less than 75% closure even at the end of eight weeks, and underwent split-skin grafting (SSG). In the conventional group, a total of 48 patients (80%) showed 75–100% closure at the end of the eighth week while the remaining 12 (four- decubitus, five- post-burn, three- post-infection) were less than 75% closed and thus required SSG [Table 2]. Therefore, although a greater number of collagen-treated wounds achieved more than 75% healing after eight weeks (52 versus 48), the difference was not statistically significant (*P*- 0.21). However, only eight collagen-treated wounds required SSG as compared to 12 wounds treated with other materials (*P*- 0.04).

Time required for complete healing did not have a linear relationship with pretreatment size of the wound. Also, wounds of the same aetiology did not show a similar healing pattern, progression of healing, appearance of granulation tissue etc. Grossly smaller wounds healed faster.

## DISCUSSION

Chronic wounds take a longer time for healing as all chronic wounds have elevated levels of matrix metalloproteinases, which result in increased proteolytic activity and inactivation of the growth factors involved in the wound-healing process. Thus, a chronic wound due to any cause is a situation that needs the use of a temporary cover for the raw surface. The use of collagen dressing has been found to inhibit the action of metalloproteinases.[4] Collagen is a biomaterial that encourages wound healing through deposition and organization of freshly formed fibres and granulation tissue in the wound bed thus creating a good environment for wound healing.[5] Collagen sheets, when applied to a wound, not only promote angiogenesis, but also enhance body’s repair mechanisms.[1,2] While acting as a mechanical support these reduce oedema and loss of fluids from the wound site, along with facilitation of migration of fibroblasts into the wound and enhancing the metabolic activity of the granulation tissue.[1,6,7] Moreover, it is easy to apply and has the additional advantage of stopping bleeding.[8] Other commonly used biological dressings include amniotic membrane and homograft skin.[9] Human amniotic membrane is easy to obtain, has a low price and provides good wound coverage and has distinct advantages compared with other biologic dressings.[9] Although the risk of transmission of viral infections e.g. hepatitis, syphilis and HIV is an important concern with the use of amniotic membrane, but with routine screening of each and every patient this risk can be easily avoided. Thus, different authors have recommended amniotic membrane strongly.[10–13] The homograft skin is another very good alternative, but causes the obvious problem of management of additional wounds. Other uses of collagen sheets in cutaneous surgery are a reasonably simple option for initial temporary coverage, and definitive reconstruction of full-thickness scalp defects created after resection of malignant tumours of the scalp.[14] As such, these may also be useful to cover the defects in oral mucosa, bones and tendons, and donor area in skin grafting procedures where large grafts are harvested.

In this study, significantly more collagen-treated wounds were rendered sterile as compared to those treated with conventional dressings, after two weeks (*P*- 0.03) and four weeks (*P*- 0.04) of treatment [Table 2]. This is due to the fact that collagen dressings cover the wound and act as an effective barrier to infection.[8] Healthy granulation also appeared significantly earlier in collagen-treated wounds as compared to conventionally treated ones (*P*-0.03). The bacterial colonization of a wound may progress to an active infection in a wound and therefore affect healing. Thus, regular surveillance of the bacterial profile and their antibiotic susceptibilities should also be a part of the overall management strategy of wound care units, so as to guide appropriate antibiotic therapy while the dressings are doing their part.[15] In the present study, this was done every three to five days or when specifically indicated.

Regarding healing of the wounds, in a study done by Veves and Sheehan[4] on 276 patients of diabetic foot ulcer divided equally into two groups, one group was treated with collagen and the second with other dressing materials. They found no significant difference in the completeness of healing of wounds when old wounds (>; six months old) were compared. But the healing was better in wounds of less than six months’ duration treated with collagen dressings. We found no significant difference in the number of wounds that achieved complete closure at eight weeks of either treatment (*P*-0.21). However, a significantly lesser number of collagen-treated wounds as compared to those treated by other dressings ultimately required SSG (*P*- 0.04). Thus, collagen dressing may avoid the need of SSG as it gets incorporated in the wound in most cases.

Although a subjective finding, most patients with collagen dressing reported to enjoy early and greater degree of mobilization and more comfort as compared to those who were applied honey, silver-sulphadiazine cream or providone iodine ointment etc.

Lastly, the present study has a few drawbacks. First, it is a retrospective study. The ideal scenario is to treat and compare two different wounds one with and the other without collagen dressing in the same patient in a prospective study. Also, this study did not include an important and more useful issue of the cost and availability of collagen dressings. These issues warrant further randomized studies. Furthermore, although in the ’collagen group’ SSG was needed for significantly lesser number of patients (eight compared to 12), this is based on the findings of a small number of patients. Thus this result cannot be generalized with high confidence. Therefore, the need for further randomized controlled studies that have a large number of patients, and are accurately designed has to be recognized from the present study.

## CONCLUSION

Collagen sheet dressing does not offer significant better results over conventional dressings in terms of completeness of healing of burns and chronic wounds. However, it may avoid the need of skin grafting, although this finding needs further substantiation by appropriately designed randomized studies of large groups.
